# Integrative Approach to Facilitate Fracture Healing: Topical Chinese Herbal Paste with Oral Strontium Ranelate

**DOI:** 10.1155/2017/9795806

**Published:** 2017-12-31

**Authors:** Wing-Sum Siu, Hoi-Ting Shiu, Chun-Hay Ko, Wai-Ting Shum, Ho-Nam Yu, Clara Bik-San Lau, Leung-Kim Hung, Ping-Chung Leung

**Affiliations:** ^1^Institute of Chinese Medicine, The Chinese University of Hong Kong, Shatin, New Territories, Hong Kong; ^2^State Key Laboratory of Phytochemistry and Plant Resources in West China, The Chinese University of Hong Kong, Shatin, New Territories, Hong Kong; ^3^Department of Orthopaedics and Traumatology, The Chinese University of Hong Kong, Shatin, New Territories, Hong Kong

## Abstract

Strontium ranelate (SrR) is one of the pharmaceutical agents reported to be effective on the promotion of fracture healing. This study aimed to evaluate the integrative effect of the oral SrR with a topical Chinese herbal paste, namely, CDR, on facilitation of bone healing. The* in vivo* efficacy was evaluated using rats with tibial fracture. They were treated with either CDR topically, or SrR orally, or their combined treatments. The* in vivo* results illustrated a significant additive effect of CDR on SrR in increasing the yield load of the fractured tibia. The* in vitro* results showed that neither SrR nor CDR exhibited a cytotoxic effect on UMR106 and bone-marrow stem cell (BMSC), but both of them increased the proliferation of BMSC at low concentrations. The combination of CDR at 200 *μ*g/mL with SrR at 200 or 400 *μ*g/ml also showed an additive effect on increasing the ALP activity of BMSC. Both SrR and CDR alone reduced osteoclast formation, and the effective concentration of SrR to inhibit osteoclastogenesis was reduced in the presence of CDR. This integrative approach by combining oral SrR and topical CDR is effective in promoting fracture healing properly due to their additive effects on proosteogenic and antiosteoclastogenic properties.

## 1. Introduction

One of the commonest consultations in orthopaedic clinics relates to fracture. The annual worldwide incidence of adult fractures is around 9.0–22.8 per 1000 people [1]. Patients with bone fracture require a long hospitalization time [2]. In the United States, the lower extremity fractures of adolescents and adults under 65 years old cause $1.2 billion [3]. Therefore, fractures not only reduce the social productivity, but also increase the health services utilization and socioeconomic burden [4–6].

Orthopaedic surgeries are undoubtedly effective in fixing fractures. However, the healing process thereafter is seldom concerned by healthcare professionals in hospitals and clinics. Patients are usually left unattended during hospitalization except for the inflammation control and pain management. The healing of the fracture relies on self-recovery. In fact, there are many scientific researchers working on the interventions to facilitate fracture healing, for instance, inventions of biophysical stimulations and applications of biomaterial scaffolds, as well as investigations on the efficacy of growth factors and bone morphogenetic proteins [7–10]. Nonetheless, the clinical application of these interventions is yet controversial.

There are growing evidences supporting that those pharmaceutical agents active in bone may be potential agents for the systemic enhancement of fracture repair. The off-label use of antiosteoporotic agents in complicated fractures and nonunions has been studied [11–13]; antiresorptive agents including calcitonin, bisphosphonates, estrogen, selective estrogen receptor modulators (SERMs), and the RANK ligand inhibitor are reported to increase the bone strength of osteoporotic patients [14]; bone forming agents including bone morphogenetic proteins (BMPs) [15], parathyroid hormone (PTH) [16–18], and strontium ranelate (SrR) [19, 20] have been well studied on fracture healing. Since the bone forming agents stimulate osteoblast function, they are considered more ideal to improve fracture repair than those antiresorptive agents. However, BMPs are solely delivered to the site of the fracture by being incorporated into a bone implant or autograft. PTH has been shown to increase osteosarcoma in rats [21, 22] and may increase the risk of osteosarcoma occurrence in humans [23]. On the other hand, SrR is called a “dual action bone agent” because it not only increases deposition of new bone by osteoblasts but also reduces the resorption of bone by osteoclasts [24]. Callus strength treated with SrR was found superior to that treated with PTH [25]. Nonetheless, the European Medicines Agency has recommended restricting the use of SrR for the treatment of osteoporosis because of its risk of heart problems. Up to now, notwithstanding that there are not few clinical studies that have been initiated to evaluate the effect of bone forming agents on fracture repair, their application to improve fracture healing by clinicians is still controversial.

On the contrary, facilitation of fracture healing is one of the major concerns in traditional Chinese medicine (TCM). TCM treatments on fractures have been adopted by Chinese for thousands of years and almost all the treatment regimens involve topical applications of herbal pastes. Nonetheless, the formulae of these herbal medicines are too diversified as yet. More importantly, the lack of relevant evidence-based scientific supports and the poor systemic documentation of the clinical data make them not well accepted worldwide.

Recently, we have conducted several preclinical studies to investigate the efficacy of topical herbal pastes on facilitation of fracture healing. A 6-herb paste increased the callus size and elevated the bone-specific alkaline phosphatase activities in a rabbit tibial fracture model. Its extract also significantly increased the proliferation of UMR106 cells and reduced the nitric oxide production in murine macrophage [26]. This paste was then simplified to a 4-herb paste containing Carthami Flos, Dipsaci Radix, Notoginseng Rhizoma, and Rhei Rhizoma, namely, CDNR. An* in vivo* topical CDNR treatment on the drill-hole defect of rat resulted in a higher yield load and work done than the control [27]. The CDNR was then further optimized to a 3-herb formula: CDR (Notoginseng Rhizoma was excluded). A clinical trial on the efficacy of the CDR herbal paste in the treatment of the fifth metatarsal fracture has been conducted recently [28]. The results showed that the pain was soothed after two weeks of treatment. The fracture sites swelling had 20% reduction in thickness measured with an ultrasonic tool. Foot and ankle functional scores were markedly improved after six weeks. Importantly, the radiological examinations revealed the early perfect fracture unions.

Considering that CDR herbal paste exhibits bone forming property, synergistic effect on promotion of fracture healing may be expected when it is cotreated with an oral medication of a bone forming pharmaceutical agent. SrR was considered in the current study because of not only its positive results on fracture healing as stated above but also its potential synergistic effect in the treatment of osteoporosis with another bone forming pharmaceutical agent [29]. If the synergistic effect of CDR and SrR is observed, the normal dosage of SrR could be reduced and hence its adverse effects could be minimized.

The current study aims to verify the efficacy of the combination use of a topical Chinese herbal paste with an oral bone forming pharmaceutical agent on promoting fracture healing through both* in vivo* and* in vitro* experiments. It also aims to provide evidence-based scientific data to support this integrative medicine approach on facilitating fracture healing.

## 2. Materials and Methods

### 2.1. Herbal Materials and Preparation of the Herbal Paste

The herbal materials used in this study were (1) Carthami Flos (*Carthamus tinctorius* L., dried flower, “Hong-Hua” in Chinese), (2) Dipsaci Radix (*Dipsacus asperoides* C.Y. Cheng T.M. Ai, dried root, “Xu Duan” in Chinese), and (3) Rhei Rhizoma (*Rheum palmatum* Linn, dried root, “Da Huang” in Chinese). All the herbs were purchased from Guangzhou Zhixin Limited (Guangzhou, China). With reference to the methods stated in the Chinese Pharmacopoeia [30], the identities of all herbs had been authenticated using thin-layer chromatography. The herbarium voucher specimens of the tested herbs were deposited in the museum of the Institute of Chinese Medicine, the Chinese University of Hong Kong, with voucher name and numbers as follows: Carthami Flos: 2013-3415; Dipsaci Radix: 2013-3417; Rhei Rhizoma: 2013-3416.

Each herb (50 g each) was extracted by reflux using 1 liter of distilled water for one hour and filtered, and the filtrate was collected. Then, the remaining solid herbal residue was further extracted by reflux using 1 liter of 95% ethanol for one hour and then filtered. The aqueous and ethanol extracts were combined and concentrated into paste form. The water content of the paste was determined. The CDR herbal paste was prepared by mixing the three individual pastes in ratio 1 : 1 : 1 (dry weight). For the* in vitro* studies, the CDR paste was weighted and dissolved in relative culture medium and then filtered by 0.22 mm filter. For the* in vivo* topical treatment, the CDR paste was supplemented with 2.0% (w/w) borneol (Alfa Aesar, Shanghai, China) additionally to increase the transdermal efficiency.

Strontium ranelate (SrR) (Protos®, Servier, France) was used for both* in vivo* and* in vitro* studies.

### 2.2. *In Vivo* Study

Animal ethics approval had been obtained from the Animal and Experimental Ethics Committee of the Chinese University of Hong Kong (CUHK) for the* in vivo* study (14/155/MIS). The Sprague-Dawley rats were supplied by the Laboratory Animal Service Centre (LASEC), CUHK. They were housed in normal standard cages at a constant temperature of 22°C with a 12-h light-dark cycle. Food and water were given ad libitum. The experimental procedures were started after 7 days of acclimatization.

A total of 36 male Sprague-Dawley rats with body weight of 353.3 ± 17.7 grams were used. The rats were anesthetized using ketamine and xylazine cocktail (im) and buprenorphine was given preoperatively for analgesic purpose (sc). Firstly, a Kirschner-wire with 1.0 mm diameter was inserted into the intramedullary canal from the anterior-intercondyloid fossa of the right tibia as internal fixation. Then, an open fracture was created at the mid-shaft of the tibia using an electric burr drill (OmniDrill35, World Precision Instrument, US). The incision on the skin was closed using suture finally. The left tibia was untreated. At the next day, the rats were randomly divided into 6 groups and treated with different regimens, respectively: they were (1) fed with distilled water without CDR paste as control (Ctrl); (2) fed with 200 mg/kg SrR without CDR paste (SrR200); (3) fed with 600 mg/kg SrR without CDR paste (SrR600); (4) fed with distilled water and CDR paste was applied (CDR); (5) fed with 200 mg/kg SrR and CDR paste was applied (CDR + SrR200); (6) fed with 600 mg/kg SrR and CDR paste was applied (CDR + SrR200). A gavage tube was used for all of the feedings. SrR was dissolved in distilled water and the concentrations were adjusted so that 2 ml SrR solutions, equivalent to the volume of distilled water, were administered orally. For Group 4 to Group 6, one gram of CDR paste was applied topically onto the fracture site. The fracture site of all animals was covered with a thin plastic sheet (Tegaderm®, 3M, USA). The treatment protocol was 6 days/week, for 4 weeks. 600 mg/kg/day SrR leads to a blood strontium concentration close to the human exposure after a therapeutic dose of 2 g/day [25].

At the end of the study, the tibiae of the rats were harvested. They were wrapped with gauze soaked with 0.9% saline, stored in zipped plastic bags, and kept in a −20°C freezer. A biomechanical 4-point bending test on the fracture site was performed using the Hounsfield material testing machine (KM25, Redhill, United Kingdom) with a 250 N load-cell. Loads at yield, maximum (ultimate), and break (failure) were recorded for analysis. Young's modulus (stiffness) was also calculated from the steepest slope of the elastic region of the strength-displacement curve.

### 2.3. *In Vitro* Study

Cell lines UMR106 and RAW264.7 were purchased from the American Type Culture Collection (ATCC, Manassas, VA, USA). Bone-marrow mesenchymal stem cell (BMSC) was isolated from the femora and tibiae of Sprague-Dawley rats as described previously [31, 32]. All cells were maintained in a 37°C incubator with 5% CO_2_ and 95% humidified air. The cells were cultured at the following conditions for the* in vitro* assays unless otherwise specified. 

*UMR106*. 100 *μ*l UMR106 with cell density 1 × 10^4^ cells/ml in DMEM growth medium (Life Technologies, USA) was seeded in each well of a 96-well culture plate. The cells were incubated overnight and the medium was then replaced by 100 *μ*l DMEM growth medium with only 1% FBS and incubated overnight again. The medium was finally replaced with 100 *μ*l DMEM containing CDR or SrR at concentrations ranging from 0 (control) to 400 *μ*g/ml. 

*BMSC*. 100 *μ*l BMSC at cell density 5 × 10^4^ cells/ml in *α*MEM growth medium (Life Technologies, USA) was seeded in each well of a 96-well culture plate. After 3 days of incubation, the original medium was replaced by 100 *μ*l of *α*MEM containing CDR or SrR at different concentrations.

*RAW264.7*. 100 *μ*l RAW264.7 with cell density 5000 cells/ml was seeded in each well of a 96-well culture plate with DMEM growth medium overnight. Then, the medium was replaced by 200 *μ*l *α*MEM growth medium supplemented with 0.0625% RANKL to induce osteoclastic differentiation. After further incubation for 2 days, the RANKL containing *α*MEM growth medium was replaced by 200 *μ*l *α*MEM growth medium containing CDR or SrR at different concentrations.

#### 2.3.1. Cytotoxic Effect on Different Cells

The cytotoxic effect of the CDR and SrR on all the UMR106 and BMSCs was tested by 3-(4,5-dimethylthiazol-2-yl)-2,5-diphenyltetrazolium bromide (MTT) assay. It was performed at 24 and 48 hours for UMR106 and BMSCs, respectively, after the culture medium had been replaced completely by the medium supplemented with the drugs at various concentrations. The cells incubated with medium without drug acted as control (Ctrl). 20 *μ*l MTT solution (5 mg/ml in PBS) was added to each well containing 100 *μ*l medium and incubated for 4 hours at 37°C. The resultant formazan product was dissolved in DMSO (200 *μ*l/well) and measured at 492 nm by a microplate spectrophotometer.

After the optimum concentrations of the CDR had been identified, the cells were incubated and cotreated with SrR at various concentrations to analyze the synergistic effect.

#### 2.3.2. Assessments on Bone Formation Properties

The bromodeoxyuridine (BrdU) assay was conducted to study the cell proliferating effect of the CDR and SrR on UMR106 and BMSCs. The assay was started 24 hours after the drugs had been added.

To measure the alkaline phosphatase (ALP) activity, 2.5 × 10^5^ BMSCs in 1 ml *α*MEM growth medium were seeded in 6-well plates. The cells were incubated for 6 days. Then, the original medium in the 6-well plate was replaced by osteogenic medium containing 200 *μ*g/ml of CDR or SrR, or 200 *μ*g/ml of CDR with 200 or 400 *μ*g/ml SrR (based on the results of the MTT and BrdU assays). The BMSCs incubated with osteogenic medium without drug acted as control (Ctrl). The cells were cultured continuously and their ALP activity was measured using a commercially available ALP assay kit (Stanbio, USA). Total protein content was determined with BCA protein assay reagent (Sigma, USA) and enzyme activities were expressed as U/mg protein.

#### 2.3.3. Assessments on Bone Resorption Property

After the RAW264.7 cells had been incubated with the CDR or SrR for 3 days, the tartrate-resistant acid phosphatase (TRAP) staining using an acid phosphatase kit (Sigma, USA) on the differentiated osteoclasts was performed to analyze the antiosteoclastogenic property of the drugs. The cells incubated with medium without drug acted as control (Ctrl).

### 2.4. Statistical Analysis

Data in* in vivo* study was represented as mean ± standard error of mean (SEM). Data in* in vitro* experiments was represented as mean ± standard deviation (SD). Comparison between groups was done by one-way ANOVA followed by Dunnett's multiple comparison test, unless otherwise specified. *p* < 0.05 was considered significant.

## 3. Results

### 3.1. Biomechanical Properties from* In Vivo* Study

**Figure 1:**
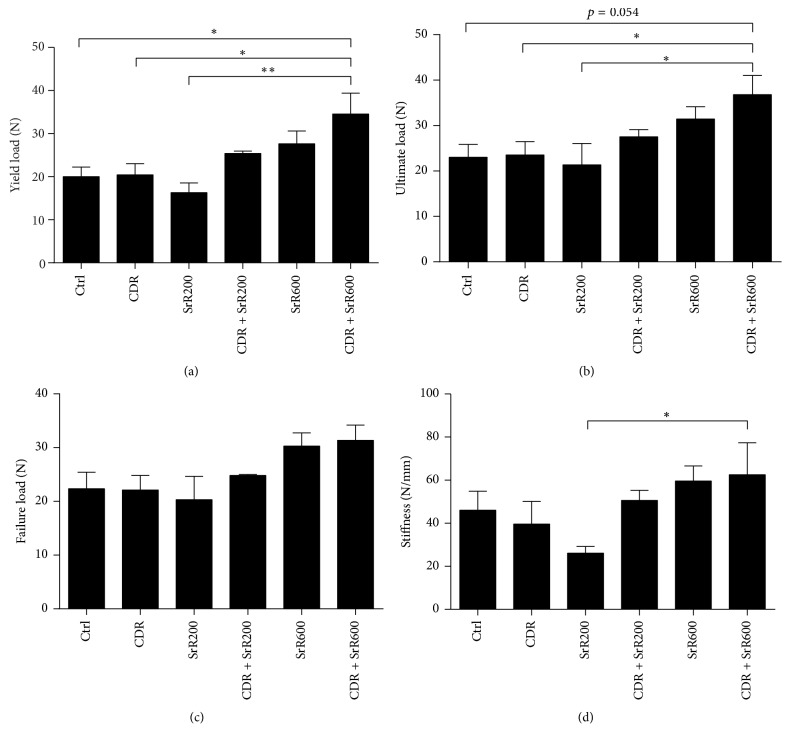
Biomechanical parameters of the 4-point bending test on the fractured tibia of rat. (a) Yield load; (b) ultimate load; (c) failure load; (d) stiffness. Ctrl: control; CDR: topical treatment of 1 g CDR; SrR200: oral administration of 200 mg/kg SrR; SrR600: oral administration of 600 mg/kg SrR. Data is expressed as mean ± SEM (error bar). ^*∗*^*p* < 0.05, ^*∗∗*^*p* < 0.01 versus CDR + SrR600 as indicated by the n-zig-zag lines.

Biomechanical 4-point bending test illustrated that the yield load of the fractured tibia of the rats which received a high oral dose of SrR (600 mg/ml) together with topical CDR application at the same time (CDR + SrR600) was significantly higher than that of the control (Ctrl) by 66.3%  (*p* < 0.05), CDR by 65.6%  (*p* < 0.05), and SrR200 by 91.2% (*p* < 0.01) (Figure 1(a)). Low oral dose SrR (200 mg/ml) or topical CDR application alone could not elevate the yield load of the fractured tibia after 4 weeks of treatment. However, their combined treatment resulted in a higher bending strength. Rats that received a high oral dose of SrR alone were also benefited with a 25.4% higher yield load compared with Ctrl, although this is not significant statistically. Similar observations were found in the analyses of the ultimate load (Figure 1(b)), failure load (Figure 1(c)), and stiffness (Figure 1(d)), except that no statistical significance was found when CDR + SrR600 was compared with Ctrl.

### 3.2. Cytotoxic Effect of CDR, SrR, and Their Combinations

**Figure 2:**
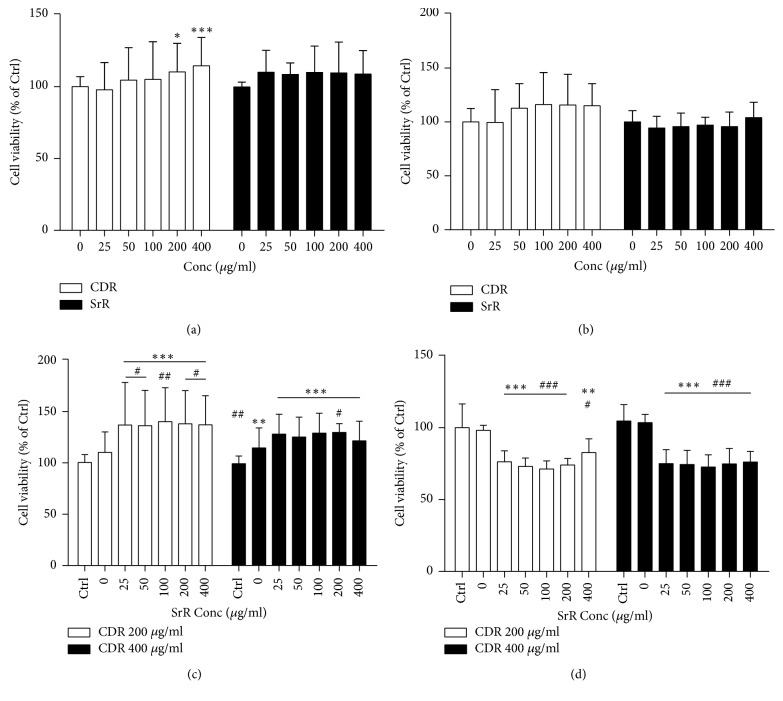
Cell viability at different concentrations of CDR or SrR assessed by MTT assay. CDR or SrR alone on (a) UMR106 and (b) BMSC, and control (Ctrl) is 0 *μ*g/ml. Cotreatment of CDR (200 or 400 *μ*g/ml) with varying concentrations of SrR on (c) UMR106 and (d) BMSC, and control (Ctrl) is the cells incubated with medium without drug (CDR and SrR). Data are expressed as mean ± SD (error bar). ^*∗*^*p* < 0.05, ^*∗∗*^*p* < 0.01, ^*∗∗∗*^*p* < 0.001 versus Ctrl; ^#^*p* < 0.05, ^##^*p* < 0.01, ^###^*p* < 0.001 versus 0 *μ*g/ml of SrR at (c) and (d). The horizontal straight line indicates the groups beneath sharing the same statistical significance.

Neither CDR nor SrR alone exhibited cytotoxic effect on UMR106 (Figure 2(a)) and BMSC (Figure 2(b)) through the MTT assay. CDR even demonstrated a significant increase in UMR106 viability from 200 to 400 *μ*g/ml by 10.3 (*p* < 0.05) to 14.6% (*p* < 0.001), compared with its control (0 *μ*g/ml). When the cells had been cotreated with CDR (at 200 or 400 *μ*g/ml) and SrR, the viability of UMR106 was enhanced significantly at all concentrations of SrR (25–400 *μ*g/ml) when compared with its plain medium control (Ctrl) (*p* < 0.001) (Figure 2(c)). In the presence of CDR at 200 *μ*g/ml, SrR could also demonstrate a significant increase in UMR viability at all concentrations when compared with CDR 200 *μ*g/ml alone (0 *μ*g/ml SrR concentration). On the contrary, the viability of BMSC was reduced in the cotreatment (Figure 2(d)) compared with the plain medium control (Ctrl) or CDR alone (0 *μ*g/ml SrR concentration).

### 3.3. Osteogenic Properties of CDR and SrR* In Vitro*

**Figure 3:**
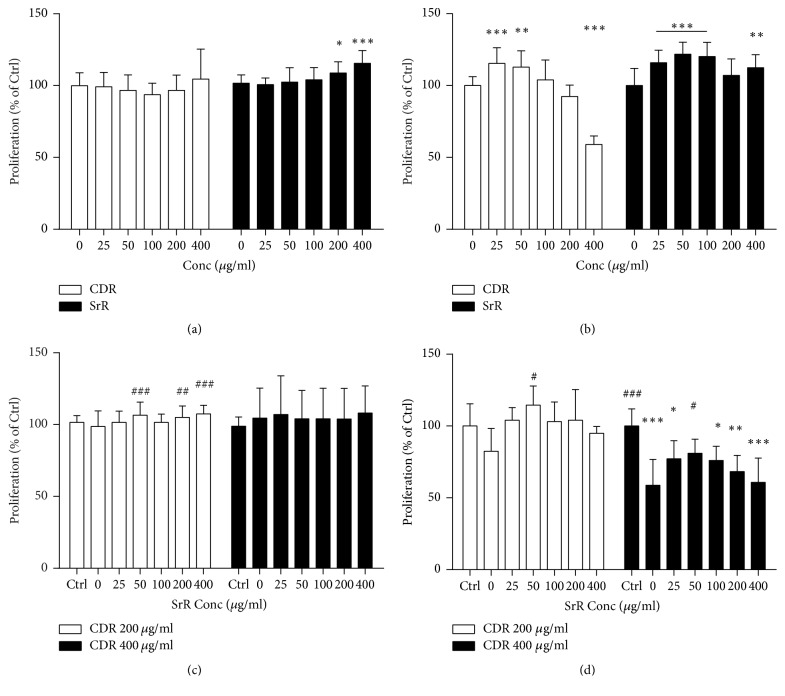
Cell proliferation at different concentrations of CDR or SrR assessed by BrdU assay. CDR or SrR alone on (a) UMR106 and (b) BMSC, and control (Ctrl) is 0 *μ*g/ml. Cotreatment of CDR (200 or 400 *μ*g/ml) with varying concentrations of SrR on (c) UMR106 and (d) BMSC, and control (Ctrl) is the cells incubated with medium without drug (CDR and SrR). Data are expressed as mean ± SD (error bar). ^*∗*^*p* < 0.05, ^*∗∗*^*p* < 0.01, ^*∗∗∗*^*p* < 0.001 versus Ctrl; ^#^*p* < 0.05, ^##^*p* < 0.01, ^###^*p* < 0.001 versus 0 *μ*g/ml of SrR at (c) and (d). The horizontal straight line indicates the groups beneath sharing the same statistical significance.

CDR alone did not promote the UMR106 cell proliferation in various concentrations in the BrdU assay but SrR did at 200 (8.8%, *p* < 0.05) and 400 *μ*g/ml (15.5%, *p* < 0.001) (Figure 3(a)). Both of them increased the proliferation of BMSC at low concentrations (Figure 3(b)). 25 and 50 *μ*g/ml CDR boosted the BMSC proliferation by 15.4% (*p* < 0.001) and 12.8% (*p* < 0.01), respectively. However, the stimulating effect on BMSC proliferation by CDR decreased as its concentration increased and a significant inhibitory effect was observed at 400 *μ*g/ml (reduced to 59.0%, *p* < 0.001) (Figure 3(b)). When compared with the plain medium without any drug (Ctrl), CDR combined with SrR at various concentrations did not demonstrate significant cell proliferative effect on UMR106 (Figure 3(c)). However, when compared with CDR at 200 *μ*g/ml alone (0 *μ*g/ml SrR), SrR at high concentrations (200 to 400 *μ*g/ml) led to a significantly higher UMR106 cell proliferation. The combined treatments with CDR at 400 *μ*g/ml showed a trend to reduce the proliferation of BMSC as the concentration of SrR increased (Figure 3(d)).

**Figure 4:**
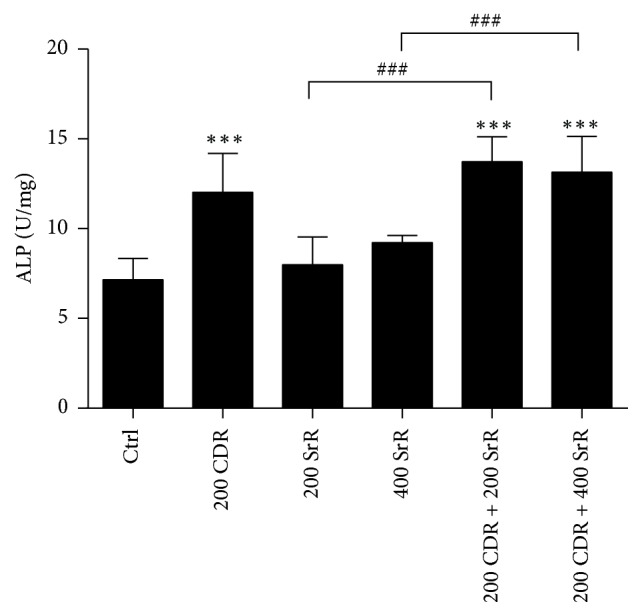
Effect of CDR and SrR on alkaline phosphatase (ALP) activity of BMSC. The numerical values in *x*-axis indicate the concentration of drug in *μ*g/ml. Control (Ctrl) is the BMSCs incubated with osteogenic medium without drug (CDR and SrR). Data are expressed as mean ± SD (error bar). ^*∗∗∗*^*p* < 0.001 versus Ctrl; ^###^*p* < 0.001 versus the group specified by the n-zig-zag lines. Comparison between groups was done by one-way ANOVA followed by Tukey's multiple comparisons test.

From the above results, the ALP activity in BMSC was analyzed at a fixed CDR concentration of 200 *μ*g/ml and SrR concentration at 200 or 400 *μ*g/ml. The result showed that CDR alone at 200 *μ*g/ml increased the ALP activity by 67.9% compared with the Ctrl (*p* < 0.001). Neither SrR at 200 nor 400 *μ*g/ml alone could elevate the ALP activity significantly. However, in the presence of CDR, SrR became effective in boosting the ALP activity of BMSC. The ALP activity increased by 91.5 and 83.4% in 200 CDR + 200 SrR and 200 CDR + 400 SrR, respectively (*p* < 0.001 both), compared with the Ctrl. Significant difference was also observed when they were compared with their respective SrR alone (*p* < 0.001 both) (Figure 4).

### 3.4. Antiosteoclastogenic Properties of CDR and SrR* In Vitro*

**Figure 5:**
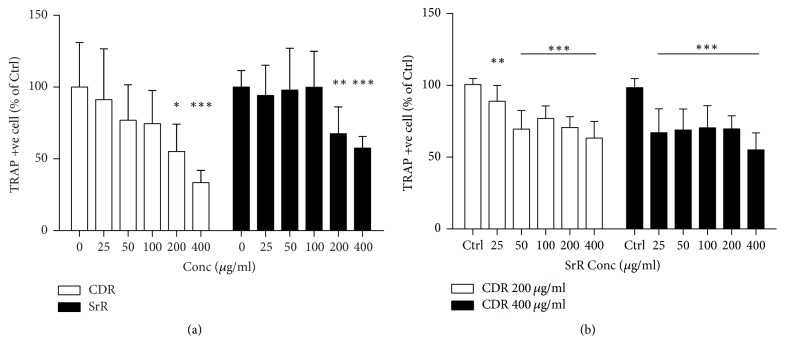
Antiosteoclastogenic effect of CDR and SrR by TRAP staining assay. (a) CDR or SrR alone, and control is 0 *μ*g/ml; (b) cotreatment of CDR (200 or 400 *μ*g/ml) with varying concentrations of SrR, and control (Ctrl) is the cells incubated with medium without drug (CDR and SrR). Data are expressed as mean ± SD (error bar). ^*∗*^*p* < 0.05, ^*∗∗*^*p* < 0.01, ^*∗∗∗*^*p* < 0.001 versus control. The horizontal straight line indicates the groups beneath sharing the same statistical significance.

**Figure 6:**
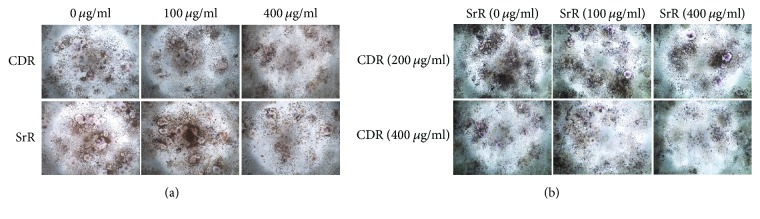
TRAP staining of RAW264.7 cells induced by RANKL. (a) RAW264.7 cells treated with either CDR or SrR individually at different concentrations (0 *μ*g/ml: plain medium). (b) RAW264.7 cells cotreated with CDR (either 200 or 400 *μ*g/ml) and SrR at different concentrations (0 *μ*g/ml: with 200 or 400 *μ*g/ml CDR only).

Both CDR and SrR alone reduced osteoclast formation starting from 200 *μ*g/ml through the TRAP staining assay (Figure 5(a)). CDR reduced the TRAP positive cell to 55.2% (*p* < 0.05) and 33.4% (*p* < 0.001) at 200 and 400 *μ*g/ml, respectively, while SrR reduced the cell number to 67.6% (*p* < 0.01) and 57.6% at 200 and 400 *μ*g/ml, respectively, compared with their blank control (0 *μ*g/ml). When CDR and SrR cotreatment was implemented, the significant effective dose of SrR to prohibit osteoclast formation was reduced to 25 *μ*g/ml (Figure 5(b)). This prohibiting effect was more obvious when the CDR concentration was increased from 200 to 400 *μ*g/ml.  Figure 6 shows the cytochemical TRAP staining of the RAW264.7 cells cultured with either CDR or SrR alone at different concentrations (Figure 6(a)) and cultured with both CDR and SrR at different concentrations (Figure 6(b)).

## 4. Discussion and Conclusion

A long history and tremendous clinical experiences in TCM on the treatments of fracture injuries exist. Among them, the topical therapy on the management of fracture healing remains crucial. However, scientific studies on topical TCM treatment for fracture healing are seldom reported in international journals. Studies in integrative approach by combining TCM and pharmaceutical agents to facilitate fracture healing are even less. This study should be the first one reporting the efficacy of an integrative medicine regimen on fracture healing.

The current study showed that the combined treatment of topical CDR with oral SrR 600 mg/kg/day resulted in the highest yield load, ultimate load, and stiffness. Biomechanical assessments are essential to evaluate the effectiveness of an intervention on fracture repair. It is because the ultimate goal of fracture repair is to restore the bone strength of the injured bone to its original level without fracture [33]. From this point of view, our results illustrated that the integrative treatment approach facilitated the fracture healing within the study period significantly. On the other hand, it is not surprising that the oral administration of 600 mg/kg/day SrR alone did not yield significant improvement on the callus strength and stiffness, even though they are higher than those of the control group. Some reports state that the oral treatment of SrR does not have a beneficial effect to increase the biomechanical properties of fracture in healthy animals [34, 35]. However, a high oral dose of SrR (450–625 mg/kg/day) did show higher mechanical strength and fracture stiffness than control group in osteoporotic animals [20, 25, 36]. In our* in vitro* study on the ALP activity of BMSC, neither SrR at 200 nor 400 *μ*g/ml alone could increase the bone formation activity of BMSC. However, once the BMSC was cotreated with SrR and CDR, this bone formation activity boosted dramatically. This illustrated the additive effect of CDR on SrR on osteogenesis and supported our* in vivo* observation that the integrative treatment regimen is effective in promoting fracture healing even in healthy animal.

Notably, the yield load and stiffness of CDR + SrR200 were comparable to those of SrR600. It indicated that when a fracture is cotreated with oral SrR and topical CDR, the dosage of SrR could be reduced without compromising the promoting effect on fracture healing. This* in vivo* finding could be explained by our* in vitro* experiments; from the MTT assay, SrR alone could not increase the cell function of UMR106 even at 400 *μ*g/ml. The BrdU assay revealed that SrR could show its proliferative effect on UMR106 at least from 200 *μ*g/ml. However, once the CDR at 200 *μ*g/ml was supplemented, SrR could increase the UMR106 viability and proliferation even at 25 *μ*g/ml (the lowest assay concentration) and 50 *μ*g/ml, respectively. From the TRAP staining assay, SrR at low concentrations (25 to 100 *μ*g/ml) could not reduce the TRAP positive cell number. Nonetheless, when CDR at 200 *μ*g/ml was supplemented, SrR exhibited the antiosteoclastogenic effect even at the lowest concentration (25 *μ*g/ml). These* in vitro* findings revealed that CDR could enhance the “dual action” of SrR and this effect was demonstrated in the* in vivo* study. In other words, the dosage of SrR for the treatment of fracture healing could be reduced so that adverse effect of SrR could, therefore, be minimized by this integrative treatment regimen.

CDR at 200 *μ*g/ml did not promote the proliferation (BrdU) of UMR106 and BMSC but greatly increased the cell viability (MTT) of UMR106. Consistently, CDR at 200 *μ*g/ml enhanced the ALP activity of BMSC. Bone-specific ALP is a key enzyme produced by osteoblasts and is recognized as a biochemical marker of bone formation. These results revealed that CDR at 200 *μ*g/ml alone did not contribute to osteoblastic cell proliferation but it increased the cell function (activity). On the other hand, when cotreated with SrR (starting from 50 *μ*g/ml), CDR at 200 *μ*g/ml became capable of promoting not only the UMR106 viability but also its proliferation (compared with CDR at 200 *μ*g/ml alone). In addition, the combination of SrR with CDR at 200 *μ*g/ml boosted the ALP activity of BMSC. All of these observations implied that the integrative treatment regimen showed an additive effect on promoting osteogenesis (enhances both osteoblastic cell proliferation and activity) which must be an important mechanism during the bone repair. It is particularly true during the reparative phase of fracture healing when endochondral ossification takes place and osteoblasts start to form new lamellar bone on the cartilaginous callus [37].

Our* in vitro* studies demonstrated that both SrR and CDR are not cytotoxic to BMSC and UMR106. Coherent observations have been reported by others. A recent report showed that at the similar concentration range and treatment period to our current study, the viability of the BMSCs isolated from ovariectomized rats treated with SrR did not have a significant difference when compared with the control group via the MTT assay [38]. SrR showed neither deleterious effect on nodule formation nor matrix mineralization* in vitro* [39]. Our previous study using CDNR (a herbal paste with addition of Notoginseng Rhizoma in CDR) also revealed that neither the ethanol nor the aqueous extract of the CDNR exhibited harmful effect on UMR106 [27]. However, the coculture of SrR and CDR at a high concentration reduced the BMSC viability. CDR at 400 *μ*g/ml alone or combined with SrR also inhibited the proliferation of BMSC. On the contrary, the coculture did not bring any adverse effect on UMR106. These observations illustrated that the combination of SrR and CDR at a high concentration reduced the proliferation of premature osteoblastic cells but promoted the cell function of mature osteoblastic cells. They also suggested that the administration of a high concentration CDR should be considered carefully if its treatment pathway is the same as SrR, that is, oral administration.

In the current* in vivo* study, CDR and SrR were applied in different pathways. The former was applied topically, while the later was served orally. The efficacy of CDR is localized while that of SrR is systemic. Although the transcutaneous efficacy of the CDR herbal paste is guaranteed, its concentration at the facture is far below its original concentration applied on the skin of the rat. In our previous transdermal study, the concentration of the chemical markers of the topical herbal paste CDNR in both the skin and muscle at the treatment sites of the rats was detected by LC/MS [27]. Although almost all of the chemical markers were detected in the tissues, the most abundant chemical marker (rhein) in the muscle was less than 0.5 *μ*g/g, which was less than 0.05% in the herbal paste. It will be expected that the concentration of the active ingredients of the herbal paste at the fracture is much less. Therefore, the* in vitro* undesirable effect on premature osteoblastic cells by the integration of SrR and CDR at a high concentration would not be expected* in vivo*.

SrR is a “dual action bone agent” because it not only increases deposition of new bone by osteoblastic activities [40, 41] and increases osteogenic differentiation of BMSC of the animal [42] but also reduces the resorption of bone by osteoclasts [43–45]. These effects could also be observed in the* in vitro* experiments of the current study. SrR increased the cell proliferation of both UMR106 (in a dose-dependent manner) and BMSC through the BrdU assay. It also reduced the osteoclast formation at high concentrations in the TRAP staining assay. These similar dual action observations have also been reported by Bonnelye and her colleagues, although they employed different cell models [24].

CDR alone also exhibited this “dual action” characteristic. It increased the cell viability of UMR106 in a dose-dependent manner via the MTT assay, promoted BMSC proliferation at low concentration through the BrdU assay, and increased the ALP activity of BMSC at 200 *μ*g/ml. It also inhibited the osteoclast formation at high concentrations in the TRAP staining assay. Our previous studies on two topical herbal formulae containing CDR also revealed that they enhanced the proliferation of UMR106 and their ethanol extracts showed an anti-inflammatory effect by suppressing the nitric oxide production in LPS-induced RAW264.7 cells [26, 27].

In conclusion, the present study clearly confirmed the* in vivo* efficacy of the integrative treatment regimen, which is the combination of topical CDR herbal paste with oral SrR, to facilitate the fracture healing on the rat tibial fracture model. This* in vivo* outcome was then explained by the* in vitro* experiments. The results elucidated that the beneficial effect of this integrative approach on fracture healing might be due to the additive effect of CDR on SrR in bone formation (proliferation and activation) as well as bone resorption. This study demonstrated for the first time the integrative intervention of East and West medications on fracture repair. It also provided a solid scientific evidence to support this integrative approach as a promising complementary treatment regimen to be considered to facilitate fracture healing.

## References

[1] Court-Brown C. M., Caesar B. (2006). Epidemiology of adult fractures: a review. *Injury*.

[2] Morrison R. S., Magaziner J., McLaughlin M. A. (2003). The impact of post-operative pain on outcomes following hip fracture. *PAIN*.

[3] MacKenzie E. J., Bosse M. J., Kellam J. F. (2000). Characterization of patients with high-energy lower extremity trauma. *Journal of Orthopaedic Trauma*.

[4] Lippuner K., von Overbeck J., Perrelet R., Bosshard H., Jaeger P. (1997). Incidence and direct medical costs of hospitalizations due to osteoporotic fractures in Switzerland. *Osteoporosis International*.

[5] Kilgore M. L., Morrisey M. A., Becker D. J. (2009). Health care expenditures associated with skeletal fractures among medicare beneficiaries, 1999–2005. *Journal of Bone and Mineral Research*.

[6] National Osteoporosis Society (2011). *25th anniversary report – A fragile future*.

[7] Shakouri K., Eftekharsadat B., Oskuie M. R. (2010). Effect of low-intensity pulsed ultrasound on fracture callus mineral density and flexural strength in rabbit tibial fresh fracture. *Journal of Orthopaedic Science*.

[8] Calori G. M., Mazza E., Colombo M., Ripamonti C. (2011). The use of bone-graft substitutes in large bone defects: any specific needs?. *Injury*.

[9] Wernike E., Montjovent M.-O., Liu Y. (2010). VEGF incorporated into calcium phosphate ceramics promotes vascularisation and bone formation in vivo. *European Cells and Materials*.

[10] Mokbel N., Naaman N., Nohra J., Badawi N. (2013). Healing patterns of critical size bony defects in rats after grafting with bone substitutes soaked in recombinant human bone morphogenetic protein-2: histological and histometric evaluation. *British Journal of Oral and Maxillofacial Surgery*.

[11] Aspenberg P. (2005). Drugs and fracture repair. *Acta Orthopaedica*.

[12] Bukata S. V. (2011). Systemic administration of pharmacological agents and bone repair: What can we expect. *Injury*.

[13] Jorgensen N. R., Schwarz P. (2011). Effects of anti-osteoporosis medications on fracture healing. *Current Osteoporosis Reports*.

[14] Lecart M.-P., Reginster J.-Y. (2011). Current options for the management of postmenopausal osteoporosis. *Expert Opinion on Pharmacotherapy*.

[15] Ong K. L., Villarraga M. L., Lau E., Carreon L. Y., Kurtz S. M., Glassman S. D. (2010). Off-label use of bone morphogenetic proteins in the United States using administrative data. *The Spine Journal*.

[16] Alkhiary Y. M., Gerstenfeld L. C., Krall E. (2005). Enhancement of experimental fracture-healing by systemic administration of recombinant human parathyroid hormone (PTH 1-34). *The Journal of bone and joint surgery. American volume*.

[17] Peichl P., Holzer L. A., Maier R., Holzer G. (2011). Parathyroid hormone 1–84 accelerates fracture-healing in pubic bones of elderly osteoporotic women. *The Journal of Bone and Joint Surgery—American Volume*.

[18] Aspenberg P., Genant H. K., Johansson T. (2010). Teriparatide for acceleration of fracture repair in humans: a prospective, randomized, double-blind study of 102 postmenopausal women with distal radial fractures. *Journal of Bone and Mineral Research*.

[19] Maïmoun L., Brennan T. C., Badoud I., Dubois-Ferriere V., Rizzoli R., Ammann P. (2010). Strontium ranelate improves implant osseointegration. *Bone*.

[20] Li Y. F., Luo E., Feng G., Zhu S. S., Li J. H., Hu J. (2010). Systemic treatment with strontium ranelate promotes tibial fracture healing in ovariectomized rats. *Osteoporosis International*.

[21] Jolette J., Wilker C. E., Smith S. Y. (2006). Defining a Noncarcinogenic Dose of Recombinant Human Parathyroid Hormone 1–84 in a 2-Year Study in Fischer 344 Rats. *Toxicologic Pathology*.

[22] Watanabe A., Yoneyama S., Nakajima M. (2012). Osteosarcoma in Sprague-Dawley rats after long-term treatment with teriparatide (human parathyroid hormone (1-34)). *Journal of Toxicological Sciences*.

[23] Tastekin N., Zateri C. (2010). Probable osteosarcoma risk after prolonged teriparatide treatment: Comment on the article by Saag et al. *Arthritis & Rheumatology*.

[24] Bonnelye E., Chabadel A., Saltel F., Jurdic P. (2008). Dual effect of strontium ranelate: Stimulation of osteoblast differentiation and inhibition of osteoclast formation and resorption in vitro. *Bone*.

[25] Habermann B., Kafchitsas K., Olender G., Augat P., Kurth A. (2010). Strontium ranelate enhances callus strength more than PTH 1-34 in an osteoporotic rat model of fracture healing. *Calcified Tissue International*.

[26] Peng L. H., Ko C. H., Siu S. W. (2010). In vitro and in vivo assessment of a herbal formula used topically for bone fracture treatment. *Journal of Ethnopharmacology*.

[27] Siu W.-S., Ko C.-H., Lam K.-W. (2015). Evaluation of a topical herbal agent for the promotion of bone healing. *Evidence-Based Complementary and Alternative Medicine*.

[28] Tse L., Cheng H., Tso C. (2015). Does Topical Agent Help Fracture Healing? A Pilot Study Using a Herbal Patch. *Open Journal of Therapy and Rehabilitation*.

[29] Blake G. M., Compston J. E., Fogelman I. (2009). Could strontium ranelate have a synergistic role in the treatment of osteoporosis?. *Journal of Bone and Mineral Research*.

[30] Chinese Pharmacopoeia Commisison (2015). *Pharmacopoeia of the People’s Republic of China*.

[31] Ko C. H., Siu W. S., Lau C. P., Lau C. B. S., Fung K. P., Leung P. C. (2010). Osteoprotective effects of *Fructus Ligustri Lucidi* aqueous extract in aged ovariectomized rats. *Chinese Medicine*.

[32] Siu W.-S., Wong H.-L., Lau C.-P. (2013). The effects of an antiosteoporosis herbal formula containing epimedii herba, ligustri lucidi fructus and psoraleae fructus on density and structure of rat long bones under tail-suspension, and its mechanisms of action. *Phytotherapy Research*.

[33] Habal M. B. (2002). Controlled bone regeneration: the ultimate process in bone repair. *Clinics in Plastic Surgery*.

[34] Cebesoy O., Tutar E., Kose K. C., Baltaci Y., Bagci C. (2007). Effect of strontium ranelate on fracture healing in rat tibia. *Joint Bone Spine*.

[35] Ibrahim M. R. M., Singh S., Merican A. M. (2016). The effect of strontium ranelate on the healing of a fractured ulna with bone gap in rabbit. *BMC Veterinary Research*.

[36] Ozturan K. E., Demir B., Yucel I., Cakici H., Yilmaz F., Haberal A. (2011). Effect of strontium ranelate on fracture healing in the osteoporotic rats. *Journal of Orthopaedic Research*.

[37] Schenk R., Browner B. D., Jupiter J. P., Levine A. M., Trafton P. G. (2003). Biology of Fracture Repair. *Skeletal Trauma: Basic Science, Management, and Reconstruction*.

[38] Guo X., Wei S., Lu M. (2016). Dose-dependent effects of strontium ranelate on ovariectomy rat bone marrow mesenchymal stem cells and human umbilical vein endothelial cells. *International Journal of Biological Sciences*.

[39] Barbara A., Delannoy P., Denis B. G., Marie P. J. (2004). Normal matrix mineralization induced by strontium ranelate in MC3T3-E1 osteogenic cells. *Metabolism*.

[40] Canalis E., Hott M., Deloffre P., Tsouderos Y., Marie P. J. (1996). The divalent strontium salt S12911 enhances bone cell replication and bone formation in vitro. *Bone*.

[41] Almeida M. M., Nani E. P., Teixeira L. N. (2016). Strontium ranelate increases osteoblast activity. *Tissue & Cell*.

[42] Peng S., Liu X. S., Wang T. (2010). In vivo anabolic effect of strontium on trabecular bone was associated with increased osteoblastogenesis of bone marrow stromal cells. *Journal of Orthopaedic Research*.

[43] Baron R., Tsouderos Y. (2002). *In vitro* effects of S12911-2 on osteoclast function and bone marrow macrophage differentiation. *European Journal of Pharmacology*.

[44] Caudrillier A., Hurtel-Lemaire A.-S., Wattel A. (2010). Strontium ranelate decreases receptor activator of nuclear factor-*κ*B ligand-induced osteoclastic differentiation in vitro: Involvement of the calcium-sensing receptor. *Molecular Pharmacology*.

[45] Takahashi N., Sasaki T., Tsouderos Y., Suda T. (2003). S 12911-2 inhibits osteoclastic bone resorption *in vitro*. *Journal of Bone and Mineral Research*.

